# The small incisions combined with interrupted buried suture blepharoplasty: flexible-rigid fixation

**DOI:** 10.3389/fmed.2024.1383937

**Published:** 2024-09-17

**Authors:** Jingjing Cao, Lingling Yan

**Affiliations:** ^1^Tianyou Hospital, Wuhan University of Science and Technology, Wuhan, Hubei, China; ^2^Hubei Provincial Hospital of Traditional Chinese Medicine, Wuhan, Hubei, China

**Keywords:** blepharoplasty, Small-incision combined with interrupted buried suture, flexible-rigid fixation, duration after blepharoplasty, shallow scars

## Abstract

**Background:**

The traditional full incision blepharoplasty is the most commonly used in Asia. However, it has significant drawbacks like long recovery period, excessive surgical marks etc. We offer a new suture idea and combine it with interrupted suture buried blepharoplasty to improve these disadvantages.

**Methods:**

In our procedure, the orbital septum is opened and separating the levator aponeurosis-the retro-orbital septum complex under this 3–5 millimeters small incision, a flexible-rigid fixation would be made: suture fixation was made to the tarsus-the complex-lower lip orbicularis oculi muscle. We interrupted bury the sutures in the uncut skin between the two small incisions.

**Results:**

This paper included 333 patients divided into small incision groups using flexible-rigid fixation (*n* = 244, 73.3%) and full incision groups using rigid fixation (*n* = 89, 26.7%). Both at 6-month and at 5-year postoperative follow-up, the satisfaction of small incision group was statistically higher than the full incision group. The overall postoperative complication rate was statistically significantly less in the small incision. The permanence was not statistically different. For Assignment of Postoperative Effort Score (PES) results, at 6 months postoperatively, the mean score was 8.29 ± 1.32 in the small incision group, 7.86 ± 1.54 in the full incision group. At 5 years postoperatively, the mean score was 7.48 ± 1.45 in the small incision group, 7.51 ± 1.73 in the full incision group. None were statistically different.

**Conclusion:**

The small incisions group achieves a higher level of patient satisfaction and more mild trauma in the surgical area, has a low complication rate, and a decent degree of durability.

## 1 Introduction

**FIGURE 1:**
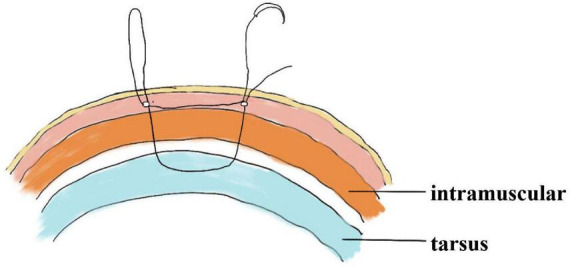
The path of the sagittal suture in interrupted buried suture blepharoplasty and anatomical structure.

Double eyelid surgery, characterized by a wide eyelid fissure, a natural appearance, and moderate scleral exposure, remains a cornerstone of aesthetic beauty in Asian cultures (1). Consequently, blepharoplasty has risen to become the most sought-after procedure in Asian plastic surgery (2, 3) Presently, blepharoplasty techniques are diverse, categorized into three primary types: nonincisional technique, the partial incisional technique, and the incisional technique (4, 5). Nonincisional technique include suture and buried thread methods. The suture method of blepharoplasty was reported by Mikamo (6) in Japan in 1896 and was the first blepharoplasty to be used as a purely aesthetic application. He proposed that the skin, orbicularis oculi muscle, levator aponeurosis, tarsus and other tissues be sutured through the blepharoplasty line to the conjunctival surface for U-shaped mattress-type sutures to form double eyelids after adhesion. And the suture buried blepharoplasty can be categorized into single continuous or multiple interrupted (7), the nylon thread is buried in the dermis-tarsus layers (Figure 1). A notable limitation of the buried line method is its relatively brief efficacy and suitability for a limited demographic with specific eye conditions like thinner skin, nonredundant or mild redundant skin, little orbicularis muscle bulk, little orbital fat (8, 9). And incision blepharoplasty, with its broad application spectrum, is widely accessible to those eligible like ptosis, loose skin, thick upper eyelid tissue etc., for blepharoplasty. Some fixation methods of incision blepharoplasty involves the formation of between the skin-tarsus adhesions to create double-eyelid, which we call a rigid fixation. Although the double eyelids formed by this fixation are long lasting and the blepharoplasty line is stable, the double eyelid formed is unnatural and the curvature is stiff. However, other fixation methods adhere to a fundamental principle: establishing a direct or indirect link between the levator aponeurosis and the skin, thereby mimicking the creation of natural double eyelids through transmission from the levator aponeurosis to the skin (10). We call this bionic methods a flexible fixation. This fixation creates a natural, graceful curve to the double-eyelid line. But the downside is that it doesn’t last very long. Initially introduced by Hayashi (11). The skin is sutured directly to the tarsus after incision of the orbicularis oculi muscle, little to no tissue removal, often resulting in a less stable and somewhat bulky eyelid. In Wu (12) improved on this by removing part of the orbicularis oculi muscle, pre-tarsus tissue and orbital septum fat, and then suturing the skin-tarsus-skin, which is the classic traditional incision and suture blepharoplasty. This method creates a stable double eyelid and is a classic rigid fixation with the characteristics described above: a long-lasting double-eyelid but with a rigid curvature and heavy surgical marks. In Park (13) developed an innovative incisional blepharoplasty, termed the orbicularis oculi-levator aponeurosis fixation method which the first flexible fixation. This approach, fixation of orbicularis oculi muscle to levator aponeurosis while preserving most of the anterior tarsus tissue, can create more natural looking double eyelid, but for the cases of congenital orbicularis oculi muscle hypertrophy would lead to a “fleshy” look in the upper eyelid. Subsequent scholars, including Zirong Ye’s team in 2015 (14), have refined Park’s method. Their composite suture technique of orbicularis oculi-levator aponeurosis-tarsus creates face-to-face tissue connections, offering improved stability over the previous linear approach. Though the incision blepharoplasty described above creates a more solid double-eyelid, this method is intricate, presents challenges in suture adjustment for symmetry, risks overtightening leading to impeded microcirculation, and can prolong postoperative swelling recovery to a longer recovery time after blepharoplasty, obvious signs of surgery, and more postoperative complications, so many people have proposed partial incision blepharoplasty. In the partial incision blepharoplasty proposed by Lam and Kim (15) in 2003, one incision of approximately 1.5 cm is made in the middle of the upper eyelid where the skin is cut along with the orbicularis oris muscle. Then Lam performs a skin-orbicularis muscle fixation suture at this incision. This method reduces the length of the incision, but the design of the eyelid shape is limited and for the lack of fixation of the blepharoplasty line at the head and tail of the eye resulting in the eyelid does not last as long. A combination of incisional blepharoplasty and non-incisional blepharoplasty was then proposed, among this, Wang’s team (16) came up with the procedure that combines small incisions with interrupted buried blepharoplasty. He makes an incision of 5 mm in the skin at two places 1–3 mm from the inner and outer corners of the eye, and then makes an incision of equal length in the middle of these two incisions. Orbicularis oculi muscle-tarsus fixation sutures were made at these 3 incisions. This blepharoplasty creates a smooth double eyelid, but it does not last long and does not improve problems such as ptosis. Inspired by these developments, we propose a novel rigid-flexible connection fixation for small incision blepharoplasty, combined with interrupted buried suture blepharoplasty, as an optimal solution to these issues (17, 18).

## 2 Patients and methods

### 2.1 Study population

This study adhered to the principles of the Declaration of Helsinki and retrospectively analyzed 333 patients (male: 5; female: 328) who underwent blepharoplasty between January 2018 and July 2018. This cohort included 244 patients in the small incisions group (male: 3; female: 241), who underwent the small incisions combined with interrupted buried suture method, and 89 patients in the full incision group (male: 2; female: 87), who underwent traditional full incisional blepharoplasty. Data from patients meeting the inclusion criteria were compiled into a spreadsheet, documenting age, sex, surgery date, and follow-up. The age range was 15–54 years, with a mean age of 25.2 ± 5.8 years, including 52.6% aged 15–24 years, 40.8% aged 25–34 years, 6.0% aged 35–44 years, and 0.6% over 45 years. Personal information, surgical details, and outcomes were gathered with patient consent.

Inclusion criteria encompassed:

(1) Patients who underwent minimally invasive small incisions combined with interrupted buried suture blepharoplasty and traditional full incision blepharoplasty between January 2018 and July 2018.
(2) Patients who with single eyelid or excessively low double-eyelid crease (These are double-eyelid crease about 1–3 millimeters from the upper eyelid margin when the eyes are closed and visible only at the tail of the eye when the eyes are open.) and thin or mildly swollen upper eyelid tissue were involved.
(3) Patients who have been contacted whether online or offline to date.
(4) Patients with reasonable expectations and no mental illness.

Exclusion criteria encompassed:

(1) Prior eyelid surgery.
(2) Broken, inflammatory upper eyelid skin.
(3) Severe upper eyelid skin redundancy (Lowering the lateral third of the eyebrow and an appearance of excess skin in the lateral corner of the upper eyelid, making the eyes triangular, even with chronic blepharitis, dry eye, and misdirected lashes and other complaints which can coexist).
(4) Moderate to severe ptosis identified in preoperative examinations.
(5) Eyelid skin redundancy and swollen eyelid skin.
(6) Preference for a high crease line (above tarsus height).

Before surgery, patients were thoroughly briefed on both procedures, including the specifics, postoperative complications, and the pros and cons of each. Patients then selected their preferred method. The study included those who underwent either the small incision interrupted buried suture method or the traditional full incisional method, with follow-up criteria as follows:

(1) Consistent follow-up by the same individual, with all surgeries performed by the same surgeon.
(2) A minimum follow-up duration of 6 months.
(3) Patient is in good physical and mental health, demonstrating cooperative behavior.

### 2.2 Pre-operative preparation

Preoperatively, patients’ needs and expectations were thoroughly assessed. The desired eyelid shape was then simulated using a double eyelid shaper, considering the patient’s preferences and eye shape to determine the most suitable crease height and shape. Determine the position of the tarsus upper edge as the blepharoplasty line, which in Asians is usually made as an arc approximately 6–8 mm from the upper lid margin. The patient is instructed to look straight ahead, and the innermost part of the iris corresponds to a point on the blepharoplasty line labeled A. The outer corner of the eye corresponds to point C on the blepharoplasty line. Point A and point C are labeled point B. Then confirm with the patient that the simulated shape is satisfactory.

### 2.3 Surgical steps

#### 2.3.1 Minimally invasive small incisions combined with interrupted buried suture blepharoplasty

**FIGURE 2:**
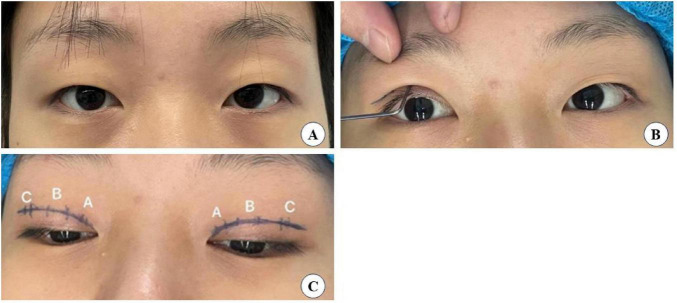
**(A)** Preoperative pictures. **(B)** Preoperative molds. **(C)** Design drawing lines.

**FIGURE 3:**
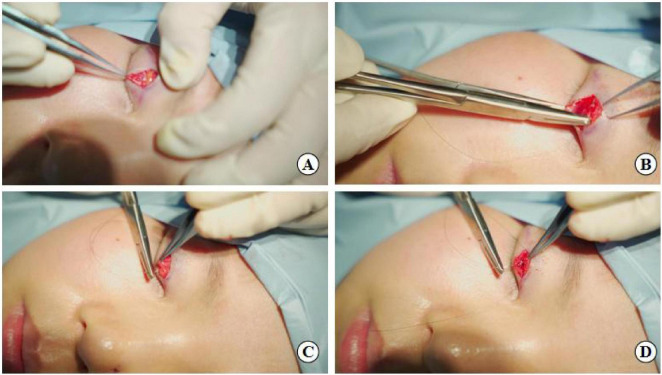
Suture fixation at point B: **(A)** Separation of the levator aponeurosis, **(B)** Hang the tarsus, **(C)** Continue to fix levator aponeurosis and orbital septum together, **(D)** Final fixation with the lower lip orbicularis oculi muscle.

**FIGURE 4:**
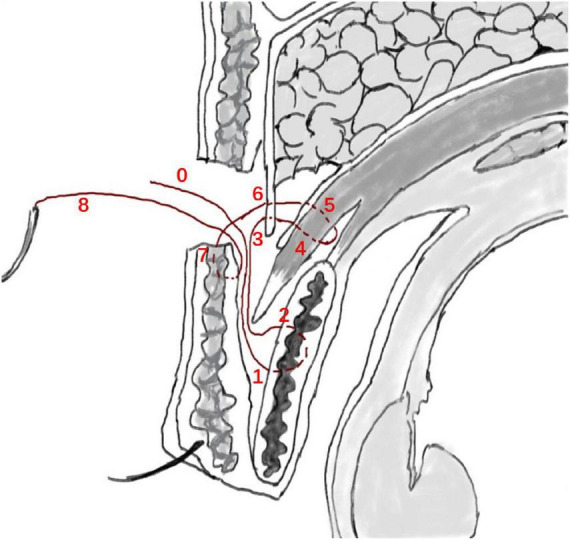
Flexible-rigid fixation: The suture is attached to the tarsus or anterior tarsus fascia, then threaded through the levator aponeurosis and the retro-orbital septum complex, and finally anchored to the orbicularis oris muscle of the lower lip using a mattress suture. Needle feed order is 0–8 through the tissue levels in sequence.

**FIGURE 5:**
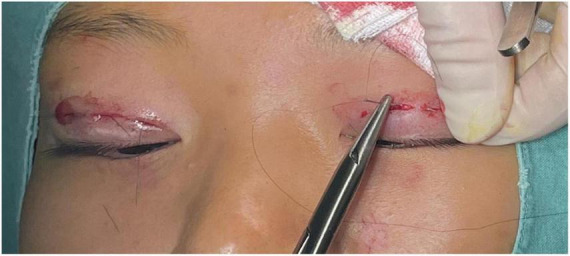
Interrupted submerged suture between two incisions.

**FIGURE 6:**
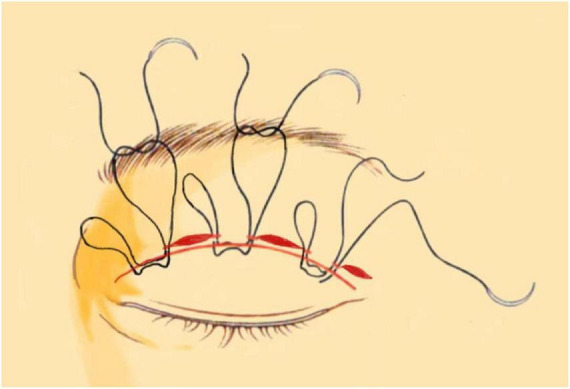
Interrupted buried suture technique under small incision: This figure shows the 6–0 nylon suture being inserted at one side of the small incision to engage the levator aponeurosis or tarsus. The suture then travels through the subcutis and exits at the opposite side of the incision.

**FIGURE 7:**
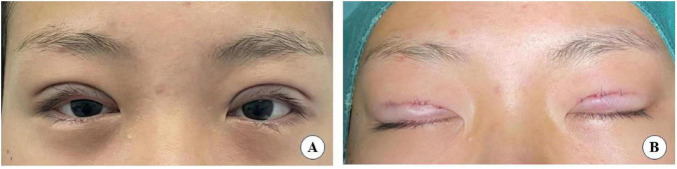
Immediate post-operative eye opening **(A)** and closing **(B)**.

**FIGURE 8:**
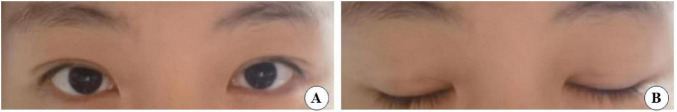
The same patient who eye opening **(A)** and closing **(B)** at 3 months after surgery.

The surgical small incision line, approximately 3–5 mm, was designed based on the preoperative simulation with the double eyelid shaper (Figure 2). This line was marked at point A above the inner canthus, point B above the upper eyelid margin’s highest point, and point C above the outer canthus. The procedure was performed under local anesthesia, following routine disinfection and administration of a local anesthetic injection containing 1% lidocaine and 1:100,000 epinephrine. Post-anesthesia, the skin was incised along the predetermined small incision lines (Figure 2C). Using ophthalmic scissors, the skin edges and orbicularis oculi muscle were trimmed, avoiding excision of the orbicularis oculi muscle at this stage. At point C, the orbital septum was opened to expose the preaponeurotic fat, allowing for the removal of herniated fat in cases of eyelid swelling. It is crucial to control bleeding during this operation using bipolar electrocoagulation or hemostatic forceps to prevent postoperative hematoma and bruising, which in severe cases could compress the optic nerve and affect vision (19). Finally, at point B, the levator aponeurosis and retro-orbital septum complex were separated. The 6–0 nylon suture is first attached horizontally to the tarsus or anterior tarsus fascia, then threaded through

the levator aponeurosis and the retro-orbital septum complex. The needle is subsequently reversed to pass again through these structures, incorporating the lower lip of the orbicularis oculi muscle to create a mattress suture (Figures 3, 4). Employing this suture fixation technique, 2–3 stitches are placed, and the patient is asked to open and close their eyes to fine-tune the shape. At points A and C, we employed the classic skin-tarsus-skin rigid fixation. Therefore, the overall suture approach at points A, B, and C remains flexible-hard suture fixation. The flexible fixation involves suturing the retro-orbital septum and levator aponeurosis complex to the orbicularis oculi muscle of the lower lip, while the rigid fixation entails suturing the skin to the tarsal plate. This technique is a modification of Park’s method (20, 21), fixation sutures are both placed through the orbicularis oculi muscle-lid, instead of direct skin-tarsus fixation. Inspired by this we separated the levator aponeurosis and sutured it with the orbital septum to form a tarsus-levator aponeurosis-orbital septum-orbicularis oculi complex. Since the orbicularis muscle is tightly bound to the skin, it forms an indirect connection between the tarsus and the skin, increasing the sense of naturalness and stability of the double eyelid. Next, we intermittently bury the sutures in the uncut skin between the two small incisions. The surgeon inserts the 6–0 nylon sutures at the trailing side of the small incision (Figure 1) to engage the levator aponeurosis or tarsus, slightly lifting the eyelashes, and exits out of the head of the other incision. The needle re-enters at the original exit point, travels back along the subcutis to the original entry point, and then exits (Figures 1, 5, 6). This procedure secures the levator aponeurosis to the subcutaneous tissue in the pretarsal region. The patient is asked repeatedly to open their eyes, allowing the surgeon to adjust the depth and smoothness of the newly formed double eyelid crease to an optimal level. The final two buried lines are completed in the usual manner. In the four unincised skin areas between the three incisions, including the inner and outer eye corners, the same interrupted buried suture technique is applied. After completing the buried lines, the skin at the small incision is closed with 8–0 nylon thread using counter stitching. The contralateral sides are treated symmetrically, and upon completion of the surgical procedures, the disinfected area, previously stained with iodine, is cleaned with saline (Figures 7, 8).

#### 2.3.2 Traditional full-incision blepharoplasty

The height and shape of the eyelid fold are marked using a marker, based on the simulation by the preoperative eyelid shaper. The extent of skin removal is tailored to the laxity of the upper eyelid skin. Routine preparation includes laying a disinfectant towel and administering 1% lidocaine with 1:100,000 epinephrine as a local anesthetic. Post-anesthesia, the skin and a proportionate amount of prelid tissue are excised according to the preoperative plan and the degree of upper lid swelling. The orbicularis oculi muscle of the lower lip is sutured to the tarsus using 7–0 nylon suture at key points: the inner and outer corners of the eye, the highest point of the upper lid margin, and two midpoints between these three locations. This suture method, connecting directly to the tarsus, is termed rigid fixation (22). The patient is then asked to sit up and open their eyes, allowing for adjustments to the eyelid fold to achieve optimal results. The skin at the upper lid incision is smoothly aligned and closed with 8–0 nylon sutures.

### 2.4 Postoperative care and evaluation

Immediately after surgery, the area is wrapped in gauze under pressure, which can be removed after 24 h. The surgical incision should not be exposed to water until suture removal, during which it can be cleaned with saline or 0.5% iodine. Sutures are removed at our outpatient clinic 5–7 days post-surgery. Patients are advised to open their eyes frequently and to refrain from rubbing their eyes or applying makeup for one month post-surgery.

#### 2.4.1 Follow-up study and investigation

The follow-up period ranged from six months to five years post-surgery. Patient information, including name, gender, age, and surgery type, was obtained from surgical records. All patients were consistently followed up by the same medical staff via telephone. Patient satisfaction levels (satisfied, somewhat satisfied, unsatisfied) were assessed at six months and five years post-surgery during the interviews. Responses of “satisfied” and “somewhat satisfied” were collectively categorized as satisfactory, while “unsatisfied” responses were considered unsatisfactory. Unsatisfied patients were instructed to take photographs of their eyelids and send them to the same medical staff for documentation of unsatisfactory surgical outcomes and complications.

#### 2.4.2 Assignment of Postoperative Effort Score (PES)

Some creases may fade over time, while others may form only partially form across the palpebral fissure. The success of the procedure heavily relies on the surgeon’s expertise in determining the appropriate crease height, ensuring its optimal depth for a dynamic appearance, and shaping it accurately. Four crucial factors must be considered: height (H), shape (S), continuity (C), and permanence (P) (23). Therefore, we developed an in-house scoring system, the Assignment of Postoperative Effort Score (PES), to assess patients in five aspects—S, H, C, P, and others—during telephone follow-ups. The scoring criteria encompass five primary categories: shape (S), height (H), continuity (C), permanence (P) and others (O), with each category scored out of two points based on specific criteria:

1. Shape (S): 2 points

(a) Asymmetry
(b) Exaggerated lateral flare/Semilunar crease, or other undesirable double eyelid crease shapes

2. Hight (H): 2 points

(a) Incorrect height (too high or too low) due to erroneous incision marking, suture placement, or edema
(b) Excessive fat removal leading to a high sulcus

3. Continuity (C): 2 points

(a) Fragmented or discontinuous crease
(b) Presence of multiple creases

4. Permanence (P): 2 points

If the eyelid crease is completely detached, this item scores 0.

(a) Partial or complete obliteration
(b) Shallow crease

5. Others (0): 2 point

(a) Complications
(b) Miscellaneous issues

The text continues here.

### 2.5 Statistical methods

Data processing was conducted using SPSS 26.0 and GraphPad Prism statistical software. Measurement data are presented as means ± standard deviation (SD) and compared within groups using the Mann–Whitney U test. Statistical data are expressed as [*n* (%)], and analyzed using the χ^2^ test, Yate’s correction for continuity, Fisher’s exact test. A *P*-value < 0.05 was considered indicative of a statistically significant difference.

## 3 Results

### 3.1 Follow-up results: patient satisfaction and complication rate survey

Among the 333 participants in this study, 328 (98.5%) were female, and 5 (1.5%) were male. The demographic data are presented in Table 1. The mean age did not significantly differ between the groups (24.7 ± 5.0 years versus 26.4 ± 7.4 years, *p* = 0.308), nor did the male-to-female ratio (3/241 vs. 2/89, *P* = 0.868). Thus, it can be inferred that age and gender did not significantly impact the outcomes in the experimental and control groups.

**TABLE 1:** General information about the patient.

	Small incisions group (*n* = 244)	Full cut group (*n* = 89)	*P*-value
Age (mean ± SD)	24.7 ± 5.0	26.4 ± 7.4	0.308
Sex (male/female ratio)	3/241	2/89	0.868

Postoperative patient satisfaction and complications are detailed in Tables 2–4. Six months post-surgery, the small incisions group reported 182 very satisfied, 51 somewhat satisfied, and 11 unsatisfied patients. In contrast, the full incision group had 56 very satisfied, 21 satisfied, and 12 unsatisfied patients at the same postoperative interval (Table 2). The satisfaction rate was higher in the small incision group compared to the full incision group (95.5 vs. 86.5%, *p* = 0.004), indicating a significant difference (Table 3). At the 5-year follow-up, the small incision group had 125 satisfied, 94 somewhat satisfied, and 25 unsatisfied patients. For the full incision group, these numbers were 49, 22, and 18, respectively (Table 3). The long-term satisfaction rates (89.8 vs. 79.8%, *P* = 0.016) also significantly differed between the groups. Therefore, it is evident that patient satisfaction in the small incision group is considerably higher than in the full incision group, both in the short and long term.

**TABLE 2:** Number of satisfied patients after surgery.

	Six months after surgery			Five years after surgery		
	Satisfied	Somewhat satisfied	Unsatisfied	Satisfied	Somewhat satisfied	Unsatisfied
Small incisions group	182 (74.6%)	51 (20.9%)	11 (4.5%)	125 (51.2%)	94 (38.5%)	25 (10.3%)
Full incision group	56 (62.9%)	21 (23.6%)	12 (13.5%)	49 (55.1%)	22 (24.7%)	18 (20.2%)

**TABLE 3:** Comparison of surgical results.

Surgical results	Small incisions group (%)	Full incision group (%)	*P*-value
Satisfaction at six months after surgery	233 (95.5%)	77 (86.5%)	0.004
Satisfaction at five years after surgery	219 (89.8%)	71 (79.8%)	0.016
The total incidence of complications	30 (12.3%)	32 (36.0%)	< 0.001

**TABLE 4:** Comparison of the incidence of postoperative complications between the two groups.

	Small incisions Group (A) *n*_1_ = 244	Full incision Group (B) *n*_2_ = 89	Percentage (%) A/*n*_1_*100	Percentage (%) B/*n*_2_*100	*P*-value
Recurrence	19	3	7.8	3.4	0.151
Flesh stripe sensation	0	9	0	10.1	< 0.001
Asymmetry	6	9	2.5	10.1	0.007
Protruding nodes	3	0	1.2	0	0.567
Others (below)	2	6	0.8	6.7	0.007
Upper lid depression	1	1	0.4	1.1	1.000
Overly wide/narrow double eyelids	1	1	0.4	1.1	1.000
Keloidal hyperplasia	0	3	0	3.4	0.019
Non-fluent double eyelid crease	0	1	0	1.1	0.267
Total Complication	30	32	12.3	36.0	< 0.001

Comparison regarding the incidence of postoperative complications and statistical analysis of variance between the two groups can be seen in Table 4. Among the groups with *P* < 0.05 were the following: the occurrence of flesh-striping sensation (*p* < 0.001), asymmetry in double eyelid lines (*p* = 0.007), other complications including keloidal hyperplasia (*p* = 0.019), and the overall complication rate (*p* < 0.001) were significantly lower in the small incision group than in the full incision group. However, issues such as recurrence, protruding line knots, and non-fluent double eyelid creases did not show significant differences between the two groups.

### 3.2 Assignment of Postoperative Effort Score (PES) results: physician scoring and statistical analysis based on follow-up results

**FIGURE 9:**
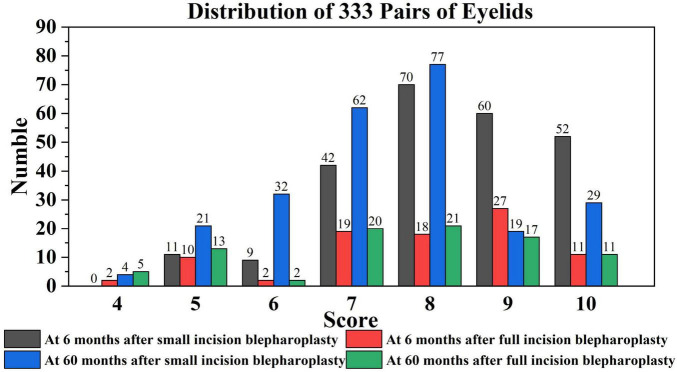
This chart illustrates the distribution of PES scores among post-operative blepharoplasty patients in each group, according to the PES scoring rules.

**FIGURE 10:**
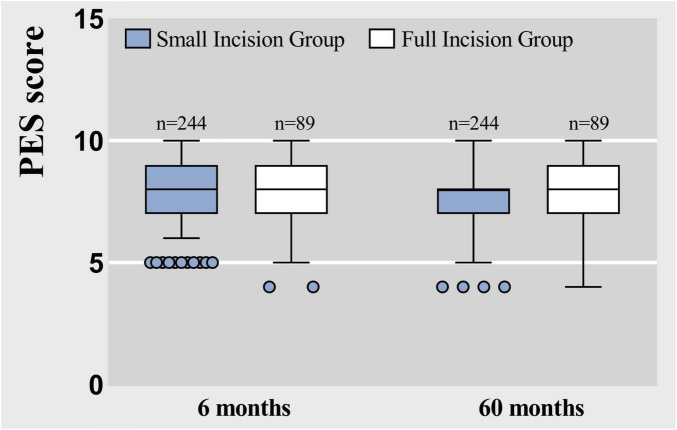
Box plots represent raw data from the Postoperative Effort Score (PES) at 6 months and 60 months, showing the median (centerline), interquartile range (box margins), adjacent values (whiskers), and outliers (dots).

Follow-up assessments for each patient were conducted and scored by the same physician, ensuring an objective and impartial evaluation based on the Postoperative Effort Score (PES) criteria, with the scores summarized as shown in Figures 9, 10. The results in Table 5, which indicated that at 6 months post-surgery, 233 patients in the small-incision group and 77 patients in the full-incision group achieved PES scores of 6 or higher. At the 5-year mark, 219 patients in the small-incision group and 71 in the full-incision group maintained PES scores of 6 or higher, aligning with the patient satisfaction survey findings. Regarding the mean PES scores, at 6 months postoperatively, the small incision group had a score of 8.29 ± 1.32, and the full incision group scored 7.86 ± 1.54, (*P* = 0.052), showing no statistical difference. At 5 years postoperatively, the mean scores were 7.48 ± 1.45 for the small incision group and 7.51 ± 1.73 for the full incision group (*P* = 0.504), also indicating no statistical difference.

**TABLE 5:** Analyses of 6 months and 5 years PES score (imputed) outcome.

	6 months		5 years	
	Small incision group	Full incision group	Small incision group	Full incision group
≥ 6 score	233	77	219	71
< 6 score	11	12	25	18
Mean	8.29 ± 1.32	7.86 ± 1.54	7.48 ± 1.45	7.51 ± 1.73
*P*-value	0.052		0.504	

## 4 Discussion

In this study, the small incision group consisted of patients who underwent small incision blepharoplasty with flexible-rigid connection sutures, while the control group consisted of patients who underwent full incision blepharoplasty with rigid fixation.

**FIGURE 11:**
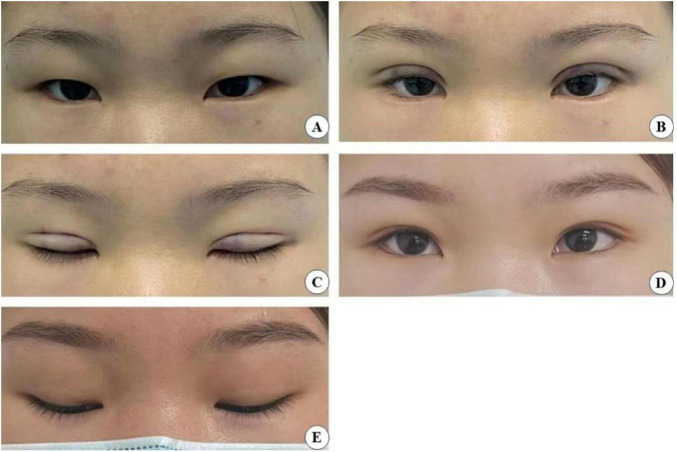
A 24-year-old female patient who underwent minimally invasive small incision combined with interrupted buried blepharoplasty is shown before surgery **(A)**, immediately after surgery with open **(B)** and closed **(C)** eyes, and four years post-surgery with open **(D)** and closed eyes **(E)**.

**FIGURE 12:**
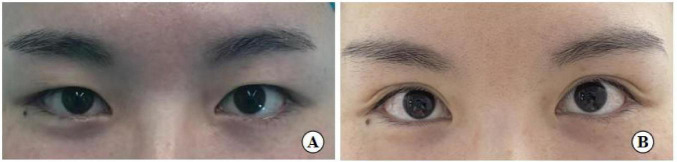
A 26-year-old female patient with good eye condition who underwent the small incision blepharoplasty is shown before surgery **(A)**, five years post-surgery **(B)**.

**FIGURE 13:**
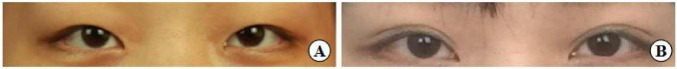
A 31-year-old female patient with moderately bloated upper eyelids and mild ptosis who underwent the small incision blepharoplasty is shown before surgery **(A)**, seven years post-surgery **(B)**.

**FIGURE 14:**
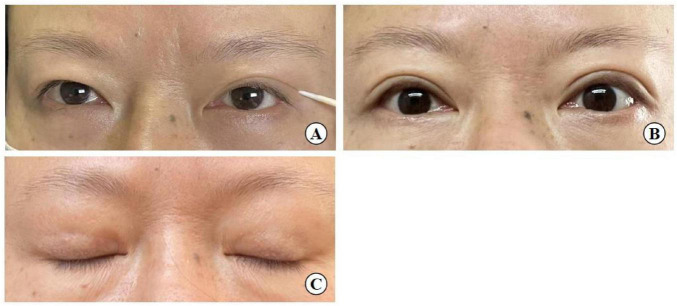
A 54-year-old woman with mild upper eyelid skin redundancy. **(A)** Pre-operative design (left eye with double eyelid formed by the action of a toothpick). **(B)** Eye opening six months after surgery. **(C)** Eye closing six months after surgery.

Our analysis revealed that the small-incision group (the experimental group cases are shown in Figures 11–14) demonstrated significantly better postoperative outcomes than the full-incision group, both in the short and long term. Additionally, the incidence of postoperative complications was markedly lower in the small-incision group than in the total-incision group. The reasons for these outcomes are as follows:

(1) The procedure in the small-incision group involved not only rigid fixation to the tarsus, as seen in the full incision group, but also flexible fixation using the levator aponeurosis as an anchoring point. The double eyelid shape was more natural in the small incision group compared than the traditional incision method, and there was no significant difference between the two groups in the rate of recurrence. So to summarize the comparison the small incision is more acceptable for who with single eyelids and not too bad eye conditions.
(2) The minimally invasive small incision technique, when combined with interrupted suture blepharoplasty, achieves both face-to-face adhesion at the incision sites and linear adhesion at non-incision areas. This point-line-face fixation strategy enhances the stability of the eyelid fold.
(3) The fixation suture involving the tarsus-levator aponeurosis and orbital septum complex-orbicularis oris muscle at point B forms a closed loop above the incision. This structure prevents sagging of aponeurotic fat, ensuring the longevity and aesthetic quality of the blepharoplasty, and also prevents additional adhesions of orbital septum tissue with surrounding tissues, reducing the likelihood of multiple eyelid formation.
(4) Diverging from the traditional full incision approach, which fully exposes the upper eyelid tissue structure by opening the skin, our method involves three 3–5 mm surgical incisions. These allow for precise manipulation during double-eyelid crease fixation. This technique, however, is not recommended for patients with moderate to severe ptosis due to its limited corrective capacity, although it can effectively address mild ptosis. In this surgery, under small incisions combined with interrupted buried suture blepharoplasty was shown to improve mild skin laxity, similar to the “quilt folding” effect, where the excess skin is folded underneath the buried suture (Figure 14). But it doesn’t remove too much excess skin.

## 5 Conclusion

In conclusion, small incision blepharoplasty combined with interrupted buried sutures offers advantages over the traditional full incision method, including a shorter recovery period, more natural-looking folds, and fewer complications. The durability of the double-eyelid line is comparable to that achieved with full incision techniques. The flexible-rigid fixation method contributes to a more stable and natural blepharoplasty line.

## Data Availability

The original contributions presented in this study are included in this article/supplementary material, further inquiries can be directed to the corresponding author.
